# 

**Published:** 1892-08-13

**Authors:** 


### CONTENTS.

PAGE

**Bacteriology and the Medical Art** . . . . . . . 321

**Passing Topics.**—House Governor or Medical Superintendent ? I. 322

**The Doctor's Armchair.**—Specialism and Individualism . 323

**The Attitude of Great Writers towards Disease. IX.**—Dickens—His Medical Science . . . . . . . . . 324

**Medical History from the Earliest Times. By E. T. Withington, M.A., M.B.Oxon. XXI.**—The Medical Profession in Rome . . . . . . . . . . . . 325

**Everybody's Page** . . . . . . . . . . . 326

**Annotations.**—How Not to Dine—The Prometheus of Gas—"Records" . . . . . . . . . . . 327

**Notes and News—Scraps and Gleanings** . . . . 328, 329

**Street Troughs and Glanders** . . . . . . . 336

PAGE

**The Practitioners' Mirror and Retrospect**—

Medicine.—The Abortive Treatment of Coryza—The Present Treatment of Simple Pleurisy . . . . . . . 330

Surgery.—Trephining for Epilepsy . . . . . . . 331

New Drugs, Appliances, and Things Medical.—Sanitas . 332

**The Institutional Workshop**—

Within the Hospitals.—The Nottingham General Hospital . 333

Hospital Opinion.—The Multiplication of Metropolitan Hospitals . . . . . . . . . . . . 335

**The History and Capabilities of Herbal Simples.** By W. T. Fernie, M D.—The Common Flag . . . . . 336

**The Student of Medicine.** — Appointments—Vacancies—Pass Lists.

### EXTRA SUPPLEMENT.—THE NURSING MIRROR.

**En Passant.**—A Holiday in Switzerland—Surrey Convalescent Home—The Mary Adelaide Nurses—The Sea-Shell Mission—Victoria Hospital, Hull—Massage—Short Items . . . cxxxvii

**The Management of Consumptive Patients. II.**—Disinfection of the Expectoration . . . . . cxxxviii

**Our Indian Letter** . . . . . . . . . cxxxix

**Appointments—Hospital Porters** . . . . . . cxl

**Presentation** . . . . . . . . . . . cxlii

**Final Examinations of Nurses at the Johns Hopkins Hospital** . . . . . . . . . . . . cxli

**Wants and Workers** . . . . . . . . . . cxlii

**Everybody's Opinion.**—"The Medicine as Before"—Male Nurses—Uniforms . . . . . . . . . . cxlii

**Notes on Novelties** . . . . . . . . . . cxliii

**For Reading to the Sick.**—From a Fellow Sufferer . . cxliii

**Off Duty.**—An Application for a Vacancy . . . . . cxliv

